# Low Dose Carbon Monoxide Exposure in Idiopathic Pulmonary Fibrosis Produces a CO Signature Comprised of Oxidative Phosphorylation Genes

**DOI:** 10.1038/s41598-019-50585-3

**Published:** 2019-10-15

**Authors:** Nancy Casanova, Tong Zhou, Manuel L. Gonzalez-Garay, Ivan O. Rosas, Hilary J. Goldberg, Stefan W. Ryter, Harold R. Collard, Souheil El-Chemaly, Kevin R. Flaherty, Gary M. Hunninghake, Joseph A. Lasky, David J. Lederer, Roberto F. Machado, Fernando J. Martinez, Imre Noth, Ganesh Raghu, Augustine M. K. Choi, Joe G. N. Garcia

**Affiliations:** 1 Department of Medicine, University of Arizona Health Sciences, Tucson, AZ USA; 2 Department of Physiology and Cell Biology, University of Nevada School of Medicine, Reno, NV USA; 3 Division of Pulmonary and Critical Care Medicine, Brigham and Women’s Hospital, Harvard Medical School, Boston, MA USA; 4 Division of Pulmonary and Critical Care Medicine, University of California San Francisco, San Francisco, CA USA; 5 Division of Pulmonary, Critical Care, Indiana University, Indianapolis, IN USA; 6 Division of Pulmonary and Critical Care Medicine, University of Washington Medical Center, Seattle, WA USA; 7 Division of Pulmonary and Critical Care Medicine, University of Michigan, Ann Arbor, MI USA; 8 Pulmonary and Critical Care Medicine Section, Tulane University Medical School, New Orleans, LA USA; 9 Division of Pulmonary and Critical Care Medicine, Columbia University Medical Center, New York, NY USA; 10 Department of Medicine, Weill Cornell Medical College, New York, NY USA; 11 Division of Pulmonary and Critical Care Medicine, University of Chicago, Chicago, IL USA

**Keywords:** Gene expression, Genetics research

## Abstract

Compelling preclinical studies indicate that low-dose carbon monoxide (CO) abrogates experimental lung fibrosis. We recently reported the results of a multicenter, double-blinded, clinical trial of inhaled CO in patients with idiopathic pulmonary fibrosis (IPF). Identifying no significantly changes in metalloproteinase-7 (MMP7) serum concentration, or secondary endpoints of physiologic measurements, hospitalization, death, or patient-reported outcomes. In the present study, we evaluated the effect of low dose CO exposure (100–200 ppm) for 12 weeks on genome-wide gene expression in peripheral blood mononuclear cells (PBMC) derived from these IPF study subjects. We conducted transcriptome profiling on 38 IPF subjects with time points available at 0, 12, and 24 weeks. Total RNA isolated from PBMCs was hybridized onto the Affymetrix Human Gene 2.0 ST Array. We identified 621 genes significantly upregulated in the 24-week CO exposed group compared with the 12-week. Pathway analysis demonstrated association with Oxidative Phosphorylation (adjusted P < 0.05). We identified a clear CO signature dominated with 23 oxidative phosphorylation-related genes (FDR <10%). We confirmed the expression of nine selected gene products using Nanostring’s nCounter analysis system. These findings suggest this signature may serve as a potential genomic biomarker for CO exposure and for potential titration of dosage to allow precision testing of therapies in future low dose CO therapeutic studies in IPF.

## Introduction

Idiopathic pulmonary fibrosis (IPF) is a relentlessly progressive interstitial lung disease characterized by the excessive formation of scar tissue in the absence of any known provocation^1^. IPF patients typically experience a progressive decline in lung function leading to a fatal respiratory failure^2^. In the absence of treatment, IPF is usually fatal within 2–3 years of the onset of symptoms. Lung transplantation is the only cure for IPF patients, but many IPF patients expire before receiving a lung transplant and only 20% to 30% of IPF patients survive 5 years after diagnosis. The approvals of Pirfenidone and Nintedanib^3,4^ provides additional, much needed therapeutic options. However, 20% of IPF patients discontinue treatment as a consequence of adverse events, and the high cost of these new drugs prohibit their wider use^5^. More sobering yet is that these costly drugs have a modest impact on disease progression and survival. Thus, IPF remains an incurable disease with a dismal prognosis and there is a continuing search for better tolerated, safer and economical treatments.

The pleiotropic biological functions of carbon monoxide (CO) include protection against oxidative injury^6,7^, inhibition of cell proliferation^8^, suppression of matrix production^9^, repression of fibrinolysis^10^, modulation of apoptosis, and inflammation, and protection against other environmental insults^11–16^. CO can bind to hemoproteins resulting in modulation signal transduction pathways affecting gene regulation that result in significant reduction of processes associated with the pathogenesis of lung fibrosis. Numerous pre-clinical studies have examined the protective effect of low CO concentrations on the lung parenchyma and vasculature. Mice exhibit an increased tolerance to lethal concentrations of oxygen (hyperoxia) with increased survival and attenuation of lung injury^12^. CO provides lung protection for lung transplantation^17^, aeroallergen-induced inflammation^18^ and lethal ischemic lung injury^10^. Low levels of CO suppress bleomycin-induced lung fibrosis and CO-exposed cells displayed impaired production of extracellular matrix proteins such as fibronectin and collagen^9^. CO (100–125 ppm) was well tolerated in patients with chronic obstructive pulmonary disease (COPD) and was shown to reduce sputum eosinophilia and improves methacholine responsiveness^19^. CO has antivaso-occlusive and immunomodulatory effects beneficial to sickle cell disease patients to prevent cardiovascular complications^20^.

The use of transcriptomic data to characterize biological effects of small molecules has become popular in drug discovery projects^21^ to determine biological effects of a drug at gene expression level. We recently completed a multicenter, double-blinded, clinical trial of inhaled CO in IPF which demonstrated the safety and tolerability of low doses (100–200 ppm) of CO in IPF^22^. However, despite modest increases in carboxy-hemoglobin (CO-Hb) blood levels, low dose CO exposure failed to significantly affect the primary study endpoint of changes in metalloproteinase-7 (MMP7) serum concentration, or secondary endpoints of physiologic measurements, hospitalization, death, or patient-reported outcomes. The aim of the present study was to determine the effect of CO exposure on genome-wide gene expression in peripheral blood mononuclear cells from room air (RA) and CO-exposed IPF study subjects.

## Methods

### Study overview

**Figure 1:**
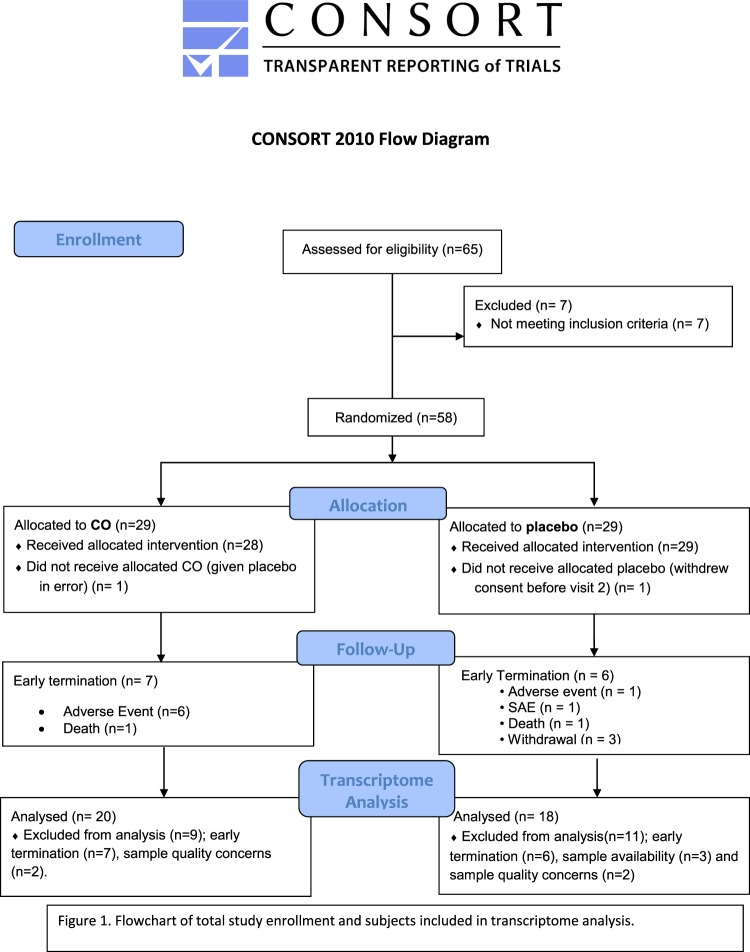
Flowchart of total study enrollment and subjects included in transcriptome and validation analysis.

We conducted a multicenter phase II randomized, double blind, placebo-controlled of clinical trial of inhaled CO compared to placebo, which was approved by each participating center’s Institutional Review Board (Brigham and Women’s IRB #210P001676). Research was performed in accordance with relevant guidelines and regulations. Written informed consent was obtained from all subjects enrolled at Brigham and Women’s Hospital-Harvard Medical School, University of Illinois, University of Chicago, University of California San Francisco, University of Michigan, Tulane University, Columbia University, and University of Washington. Participants had IPF diagnosed according to ATS/ERS/ALAT guidelines for IPF diagnosis and management^2^, mild to moderate lung disease (FVC greater than or equal to 50% predicted). Inclusion/exclusion criteria, randomization, carbon monoxide dosing and administration were previously described^22^. Briefly, subjects were randomized to inhaled CO treatment with an initial dose of 100 ppm for one week followed by dose escalation to 200 ppm, or to placebo with RA (21% inhaled oxygen). Inhaled CO was administered twice weekly for a total of 12 weeks. Peripheral blood samples were collected on specified research visits. A total of 51 subjects completed 12 weeks of randomized therapy (CO or placebo) and 45 subjects completed follow-up period. Complete enrolled cohort baseline demographics, imaging, biopsy findings and pulmonary function testing of randomized subjects were balanced; statistically significant differences were not noted between subjects treated with CO compared to treatment with placebo for any element^22^. We excluded 9 subjects in the CO arm and 11 in the RA due to early termination or sample issues, additional details are depicted in Fig. 1. There was no clinical significant differences between these and the subjects included in the gene expression analysis.

### Genomic analysis of CO trial participants

For this genomic analysis, we assessed the treatment effects in IPF subjects with at base line (week 0), end of treatment dosing period (week 12), and follow up visit (week 24). We included the subjects with isolated mononuclear cells available on these time points PBMC isolation was performed on each of the centers using the Ficoll-Paque isolation method; samples were stored at the BWH-HMS and transferred to the University of Arizona where the total RNA was isolated from PBMCs using RNAeasy MiniKit Qiagen™ following manufacturer’s protocol. RNA concentration and quality (RIN > 7) was assayed by Nanodrop™ (Thermo Fisher) and 2100 Bioanalyzer RNA™ (Agilent).

### Transcriptome profiling

RNA was hybridized onto the Affymetrix GeneChip® Human Gene 2.0 ST Array (Thermo Fisher Scientific), to conduct transcriptome profiling. The expression microarrays were analyzed using the Affymetrix Power Tools v.1.15.1. Probeset expression signals were summarized with the robust multi-array average (RMA) algorithm^23^ and log_2_ transformed with a median polish. Transcripts were considered to be reliably expressed in the samples if the Affymetrix implemented DABG (detection above ground) *P*-value was less than 0.01 in at least 67% of the samples. SAM (Significance Analysis of Microarrays)^24^, implemented in the *samr* library of the R Statistical Package, was used to compare log_2_-transformed gene expression levels between 0-week and 12-week samples and between 12-week and 24-week samples, respectively. False discovery rate (*FDR*) was controlled using the *q*-value method^25^. Transcripts with *FDR* less than 10% were deemed differentially expressed. We searched for any enriched Kyoto Encyclopedia of Genes and Genomes (KEGG)^26^ physiological pathways among the differential genes relative to the final analysis set using the NIH/DAVID^27^. An adjusted *P*-value < 0.05 after the *Benjamini-Horchberg* procedure was used as the cutoff.

### Computing geneset scores using human transcriptomic data

The Functional Analysis of Individual Microarray Expression (*FAIME*) algorithm was applied to assign a geneset score for each KEGG pathway^28^. *FAIME* computes geneset scores using rank-weighted gene expression of individual samples, which provides a translation of each sample’s transcriptomic information to molecular mechanisms^28^. Higher geneset score indicates overall upregulation of a given KEGG pathway.

### Validation

Trascriptomic results obtained by microarray were validated in the 18 subjects from the CO arm using the NanoString’s nCounter® analysis system (NanosString Technologies, Seattle, WA, USA)^29^. We selected oligonucleotides probes for molecular barcoding of the nine microarray-selected genes and three housekeeping genes (OAZ1, RPL24, RPS29). After hybridization and magnetic bead purification, barcodes were counted for each target molecule. Following normalization, we calculated the fold-change for the genes of interest. Using R Statistical Package, one tailed paired t-test (P-value < 0.05), was used to compare gene expression levels between 0-week and 12-week samples and between 12-week and 24-week samples, respectively.

## Results

### Human subjects

We conducted transcriptome analysis and validation analysis on 38 from the original 58 subjects enrolled in the clinical trial, selected based upon study completion, sample availability and sample quality Fig. 1. 20 subjects were randomized in the CO arm and 18 subjects received RA. Baseline demographics and pulmonary function testing results are outlined in Table 1. No significant differences on baseline demographic and clinical characteristics were present in the two study arms.

**Table 1:** Baseline demographics and pulmonary function testing by study arm.

Characteristics	CO (n = 20)	Room Air (n = 18)	P value
Age	65 ± 6.9	65.8 ± 8.2	0.81
Gender (Male/Female)	17(85)/3(15)	13(72)/5(28)	0.48
Race/ Ethnicity			0.11
White	15	16	
Black	2	0	
Asian	3	0	
Latino	0	2	
**PFT**			
FVC, L	3.08 ± 0.65	2.92 ± 0.96	0.435
FVC % predicted	75.6 ± 17.1	69.05 ± 13.6	0.147
TLC % predicted	70.4 ± 11	66.23 ± 13.6	0.305
Dlco % predicted	44.2 ± 11.9	41.06 ± 15.7	0.26

Values are No. (%), mean ± SD, or as otherwise indicated. CO = carbon monoxide; Dlco = diffusing capacity for carbon monoxide; FVC = forced vital capacity; TLC = total lung capacity.

### Differential gene expression induced by CO exposure

**Figure 2:**
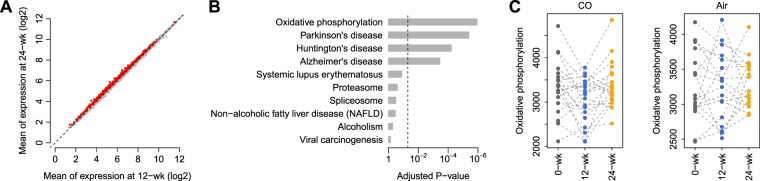
(**A**) Means of expression (log2) comparison of 621 genes significantly upregulated at 12 week and 24-week in the CO-exposed cohort. (**B)** Oxidative phosphorylation was the top KEGG pathway associated with the 621 upregulated genes (adjusted P < 0.05). A proportion of genes (40%) in this pathway overlapped with Parkinson’s disease, Huntington’s disease and Alzheimer’s disease pathways (adjusted P < 0.05). (**C)** Oxidative Phosphorylation pathway score according to FAIME algorithm indicates that the dysregulation caused by CO treatment recovers following discontinuation of CO treatment (24 wk).

At the specified significance level (*FDR* <10%), we failed to identify any differentially-expressed genes between the 0-week and 12-week groups and between the 0-week and the 24 week under CO treatment. However, 621 genes were significantly upregulated in the 24-week CO exposed group compared with the 12-week CO samples (Fig. 2A). The increase at week 24 represented a normalization to baseline values. Pathway analysis based on the KEGG database demonstrated that the top KEGG pathway associated with the 621 upregulated genes is “Oxidative Phosphorylation” (adjusted *P* < 0.05) (Fig. 2B). In addition, we found that due to the large proportion of overlapped genes (>40%) with the “Oxidative Phosphorylation” pathway; the “Parkinson’s disease”, “Huntington’s disease”, and “Alzheimer’s disease” pathways were also significantly enriched by the upregulated genes (adjusted *P* < 0.05) (Fig. 2B). We further computed the “Oxidative Phosphorylation” pathway score (see Methods for details) for both the CO and RA samples at each time point. Paired comparison revealed that for the patients with CO treatment, the “Oxidative Phosphorylation” pathway score is significantly lower at 12-week compared with 0-week (paired *t*-test: *P* = 0.035) (Fig. 2C), while this pathway is significantly upregulated at 24-week relative to 12-week (paired *t*-test: *P* = 0.009) (Fig. 2C). These results suggest that the dysregulation caused by CO treatment is remarkably recovered by discontinuation of CO treatment. As expected, patients with RA treatment did not demonstrate any significant difference in the “Oxidative Phosphorylation” pathway score among the three time points (Fig. 2C).

### Oxidative phosphorylation pathway-based gene signature

**Figure 3:**
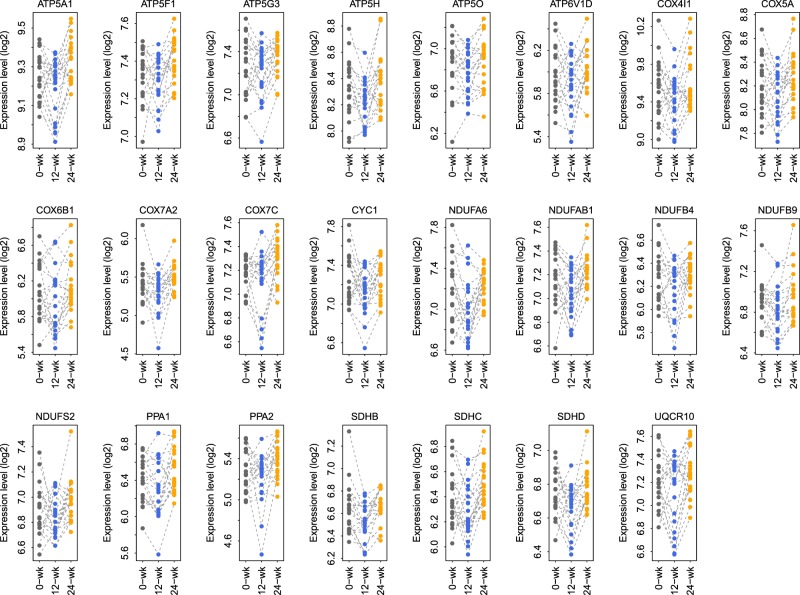
The 23 oxidative phosphorylation-related CO gene signature. Only genes within the Oxidative phosphorylation with differential expression between 12-week and 24-weeks in the CO-treated patients (FDR <10%) were retained. In total, a gene signature with 23 oxidative phosphorylation-related genes was identified.

To understand the CO-induced gene dysregulation pattern, we developed a gene signature based on the genes within the “Oxidative phosphorylation” pathway. Only the genes differentially expressed between 12-week and 24-week for the CO treated patients (*FDR* < 10%) were retained. In total, a gene signature with 23 oxidative phosphorylation-related genes was identified (Fig. 3).

### Candidate gene biomarkers confirmed by nanostring

**Figure 4:**
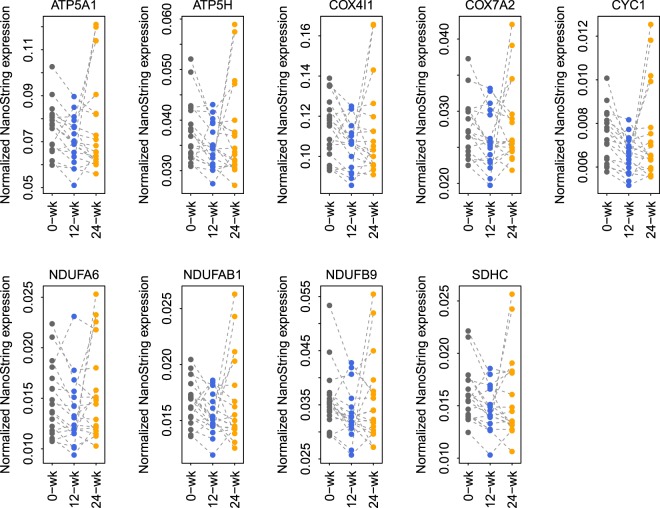
Nanostring validation confirmed the downregulation at 12-week of nine selected microarray-derived gene products (p-value < 0.05).

We confirmed the expression of nine selected gene products using Nanostring in 18 subjects from the same cohort of CO treated patients (Fig. 4). All nine genes were downregulated at 12-week compared with 0-week (one-tailed paired t-test: P < 0.05). Two genes, CYC1 and NDUFA6, were significantly upregulated at 24-week compared with 12-week (one-tailed paired t-test: P < 0.05). In the RA control group, five of the 11 genes were confirmed by Nanostring at nominal p-value < 0.05.

## Discussion

The present study identified changes in gene expression in PBMCs of Idiopathic pulmonary fibrosis patients following exposure to low dose (100–200 ppm) for 12 weeks using the Affymetrix microarray platform. A validation study was conducted in the same cohort of patients. Despite the absence of significant difference in the pulmonary function testing and functional clinical assessment in the CO-receiving group^22^, we detected significant dysregulation of gene expression in subjects receiving CO twice a week. We analyzed gene expression levels between 0-week and 12-week samples and between 12-week and 24-week samples, and 0 week and 24 week respectively. Significant differential expression was observed at week 12 and week 24 with gene dysregulation response abolished after treatment termination. The differentially-expressed genes were dominantly enriched in mitochondrial-related signaling pathways with oxidative phosphorylation, Parkinson’s disease and Huntington’s disease pathways representing the top dysregulated pathways during CO administration.

There is evidence of mitochondrial dysfunction in IPF includes a decreased efficiency of electron transport and increases in reactive oxygen species (ROS) production^30^. Oxidative phosphorylation is the coordinated transfer of electrons and protons leading to the ATP production. The mitochondrial electron transport chain (ETC) is vital for cellular energy production, but may serve as a source of aberrant ROS production from ETC complexes I and III during pathophysiological states. Complex I (NADH dehydrogenase) is a site of ROS leakage under pathophysiological conditions. CO inhibits Toll-like receptor signaling by suppressing the ROS generated through NADPH oxidase activation^29^. Mitochondria heme functional groups have been strongly implicated as a primary targets of CO action^31^. Notably, we identified that CO therapy decreased the expression of genes encoding ETC complex I genes: NDUFB4, NDUFS2, NDUFAB1, NDUFA6, NDUFB9, also enriched in Parkinson’s, Huntington’s and Alzheimer disease pathways. Similarly, complex IV, cytochrome c oxidase (COX) subunits: COX4I1 and COX7A2 within the terminal enzyme respiratory chain appear to be modulated by CO exposure with a downregulation that appear to return to baseline at week 24. CYC1, from the complex III, an ubiquinol-cytochrome c reductase complex subunit, also followed the same dysregulation pattern.

In the present study, we were able to identify the downregulation of gene expression at the mitochondrial level secondary to CO modulation at COX and NADH levels. These findings raise the possibility that CO exposure in IPF, targets modulation of mitochondrial energy production and potentially impacts mitochondrial ROS production.

Despite the reproducibility of our findings, we recognize the limitations of our study. Additional dose-titration studies are required to determine the CO dose and duration that can evoke changes on other clinical markers of disease progression. Despite these limitations, similar to other molecular profiling strategies designed to subphenotype patients with chronic lung disease^32,33^, our study suggests that a microarray-derived molecular signature successfully identifies the transcriptomic profile of CO in IPF patients dominated by significant dysregulation of genes in the oxidative phosphorylation pathway. These findings demonstrate interesting transcriptional effects associated to the CO administration and suggest this signature may serve as a potential genomic biomarker for CO exposure that can be used for dose stratification to potentially allow future precision testing of low dose CO therapies in IPF.
